# Comparative genomic analysis reveals differential genomic characteristics and featured genes between rapid- and slow-growing *non-tuberculous mycobacteria*

**DOI:** 10.3389/fmicb.2023.1243371

**Published:** 2023-09-21

**Authors:** Menglu Zhang, Peihan Wang, Cuidan Li, Ofir Segev, Jie Wang, Xiaotong Wang, Liya Yue, Xiaoyuan Jiang, Yongjie Sheng, Asaf Levy, Chunlai Jiang, Fei Chen

**Affiliations:** ^1^National Engineering Laboratory for AIDS Vaccine, School of Life Science, Jilin University, Changchun, China; ^2^CAS Key Laboratory of Genome Sciences and Information, Beijing Institute of Genomics, Chinese Academy of Sciences and China National Center for Bioinformation, Beijing, China; ^3^University of Chinese Academy of Sciences, Beijing, China; ^4^Department of Plant Pathology and Microbiology, The Institute of Environmental Science, The Robert H. Smith Faculty of Agriculture, Food, and Environment, The Hebrew University of Jerusalem, Rehovot, Israel; ^5^Key Laboratory for Molecular Enzymology and Engineering of Ministry of Education, School of Life Sciences, Jilin University, Changchun, China; ^6^Beijing Key Laboratory of Genome and Precision Medicine Technologies, Beijing, China; ^7^State Key Laboratory of Pathogenesis, Prevention and Treatment of High Incidence Diseases in Central Asia, Urumqi, China

**Keywords:** *non-tuberculous mycobacteria* (NTM), rapidly growing *mycobacteria* (RGM), slowly growing *mycobacteria* (SGM), growth rate, adaptive evolution, comparative genomics, toxin-antitoxin

## Abstract

**Introduction:**

*Non-tuberculous mycobacteria* (NTM) is a major category of environmental bacteria in nature that can be divided into rapidly growing *mycobacteria* (RGM) and slowly growing *mycobacteria* (SGM) based on their distinct growth rates. To explore differential molecular mechanisms between RGM and SGM is crucial to understand their survival state, environmental/host adaptation and pathogenicity. Comparative genomic analysis provides a powerful tool for deeply investigating differential molecular mechanisms between them. However, large-scale comparative genomic analysis between RGM and SGM is still uncovered.

**Methods:**

In this study, we screened 335 high-quality, non-redundant NTM genome sequences covering 187 species from 3,478 online NTM genomes, and then performed a comprehensive comparative genomic analysis to identify differential genomic characteristics and featured genes/protein domains between RGM and SGM.

**Results:**

Our findings reveal that RGM has a larger genome size, more genes, lower GC content, and more featured genes/protein domains in metabolism of some main substances (e.g. carbohydrates, amino acids, nucleotides, ions, and coenzymes), energy metabolism, signal transduction, replication, transcription, and translation processes, which are essential for its rapid growth requirements. On the other hand, SGM has a smaller genome size, fewer genes, higher GC content, and more featured genes/protein domains in lipid and secondary metabolite metabolisms and cellular defense mechanisms, which help enhance its genome stability and environmental adaptability. Additionally, orthogroup analysis revealed the important roles of bacterial division and bacteriophage associated genes in RGM and secretion system related genes for better environmental adaptation in SGM. Notably, PCoA analysis of the top 20 genes/protein domains showed precision classification between RGM and SGM, indicating the credibility of our screening/classification strategies.

**Discussion:**

Overall, our findings shed light on differential underlying molecular mechanisms in survival state, adaptation and pathogenicity between RGM and SGM, show the potential for our comparative genomic pipeline to investigate differential genes/protein domains at whole genomic level across different bacterial species on a large scale, and provide an important reference and improved understanding of NTM.

## Introduction

*Non-tuberculous mycobacteria* (NTM) are a major category of environmental bacteria that can be divided into rapidly growing *mycobacteria* (RGM) and slowly growing *mycobacteria* (SGM) based on their distinct growth rates (Kim et al., 2013). RGM reproduce more rapidly (3–7 days) than SGM (>7 days) in nutrient-rich environments, while SGM are better adapted to nutrient-poor and ecologically challenging environments (Pereira and Ramos, 2020). While RGM are mostly saprophytic and non-pathogenic, SGM are more pathogenic and can cause respiratory, skin and soft tissue infections, osteomyelitis and even death in severe cases (Johansen et al., 2020).

Comparative genomic analysis provides a powerful tool for deeply investigating differential molecular mechanisms between RGM and SGM (Jia et al., 2021). In recent years, there have been some studies concerning the genomic differences between RGM and SGM, with an increasing focus on exploring the mechanisms regulating the growth rates and associated pathogenic genes (Bachmann et al., 2019). For instance, several studies have shown that the RGM genomes contain more ribosomal operons and metabolic genes, aiding in faster gene expression and metabolic activities (Turenne, 2019). In contrast, the SGM genomes show the enrichment of DNA repair and oxidation–reduction reaction related genes for survival under low-nutrient conditions (Pereira and Ramos, 2020). Another study covering 157 species showed that RGM is more abundant in genes related to pathways such as Amino Acid Transport/Metabolism and Transcription, which may also be responsible for its faster growth rate (Bachmann et al., 2019). However, the study’s comprehensiveness and robustness require further enhancement, as the annotation methods were exclusively rely on the COG database.

To date, although current genomic research on limited NTM bacterial species and strains revealed some differential molecular mechanisms between RGM and SGM, large-scale comparative genomic analysis between them is still uncovered. Therefore, it is necessary to explore the genomic differences between RGM and SGM using more NTM strains and species for comprehensively analyzing the molecular mechanisms underlying the differential growth rates, environmental adaptation and pathogenicity between RGM and SGM.

In this study, we screened 335 high-quality, non-redundant NTM genome sequences covering 187 species from 3,478 online NTM genomes (as of January 2022), and then performed a comprehensive comparative genomic analysis using multiple bioinformatic tools (such as COG, Pfam, and OthoFinder). Our results comprehensively revealed differential genomic characteristics and featured genes/protein domains between RGM and SGM. The findings of our systematical analysis shed light on differential molecular mechanisms in growth, adaptation and pathogenicity underlying RGM and SGM, and provide an important reference and improved understanding of NTM.

## Materials and methods

### Collecting NTM datasets for comparative genomic analysis

A total of 3,478 Mycobacterium sequences, excluding the *Mycobacterium tuberculosis* complex and *Mycobacterium leprae* complex, were downloaded from the NCBI-genebank database,^1^ collected from January 2022. To ensure high quality and non-redundant genome datasets, strict quality control protocols were followed (Levy et al., 2018). Firstly, we calculated the N50 of genomic sequences using assemble-stats, and only those with an N50 of ≥50,000 bp were selected. Next, CheckM was used to assess the completeness and contamination of genomic sequences. Only those with a completeness score of ≥95% and contamination score of ≤5% passed the screening. Thirdly, we used Mash to calculate genomic distances and applied Markov Clustering to cluster the data. Only sequences with genomic distances of >0.01 were selected, and the redundant sequences were filtered out. Fourthly, the phylogenetic tree was aligned to remove sequences with abnormal evolutionary branches. Finally, information such as the growth rate and species classification of the genomes was manually collected from public databases (such as NCBI) and publications (Gupta et al., 2018; Matsumoto et al., 2019), resulting in 335 genomic sequences containing 187 NTM species.

In particular, we reconstructed the phylogenetic tree by extracting amino acid sequences of 198 single-copy homologeous genes detected by OrthoFinder. Single-copy gene alignment was conducted using Muscle v5.1 (Edgar, 2021), while FastTree v2.1.11 was used to generate the maximum likelihood tree (Price et al., 2009), which was then visualized using iTOL. Additionally, pairwise ANI was calculated using pyani 0.2.12 with ANIm.

### Genome characterization and gene category enrichment analysis

To analyze the genome characterization and gene category, we first predicted the genome open reading frames with Prokka v1.14.6 (Seemann, 2014). Next, we separately counted the genome size, GC content, and the number of genes annotated by Prokka with a custom Python script. Independent t-tests and PhyloGLM tests were used to compare the genome size and gene number of RGM and SGM. On the other hand, Wilcox test and PhyloGLM test were used to compare the GC content of the genome (Ives and Garland, 2010). The comparative kernel density was then plotted using the geom_density function of the R ggplot2 package.

We mapped protein-coding genes into COG ids using RPS-BLAST with an e-value threshold of 0.01 and a coverage threshold of at least 70%. For every genome, we computed the number of genes for each gene category and used t-tests and PhyloGLM to estimate the enrichment and depletion of 21 gene categories between RGM and SGM.

### Identification of gene clusters and protein domains associated with RGM and SGM

To identify gene and protein domain clusters associated with RGM and SGM, we divided up the process into two primary steps. The first involved gene and protein domain clustering based on amino acid similarity. Secondly, we identified protein and protein domain clusters that were significantly enriched in RGM/SGM. The clustering of gene and protein domains utilized three independent methods, COG, Pfam, and OthoFinder. We performed COG annotation as mentioned, with Pfam domains predicted by PfamScan v1.6 (Song et al., 2018). OrthoFinder encompassed a wider range of proteins as a clustering method, including any proteins that lacked functional annotation (Emms and Kelly, 2015). The aforementioned process generated a list of gene/protein domains for each genomic sequence. Next, we identified the genes and protein domains that were significantly enriched in RGM/SGM using Scoary (Brynildsrud et al., 2016), which is a strictly accurate statistical method with the input dataset being the presence/absence dataset of genes. These gene clusters/protein domains were considered enriched only when Benjamini-Hochberg FDR-corrected *p*-values were less than 0.05, while empirical *p*-values were less than 0.05. Identified odds ratios >1 corresponded to RGM-associated gene cluster/protein domains. In contrast, odds ratios for SGM-associated gene cluster/protein domains were < 1. Each of the 335 assembled genomes were analyzed by PHASTER (https://phaster.ca/) to identify the presence of prophage.

## Results

### Phylogenetic analysis and taxonomy of NTM genomes

**Figure 1:**
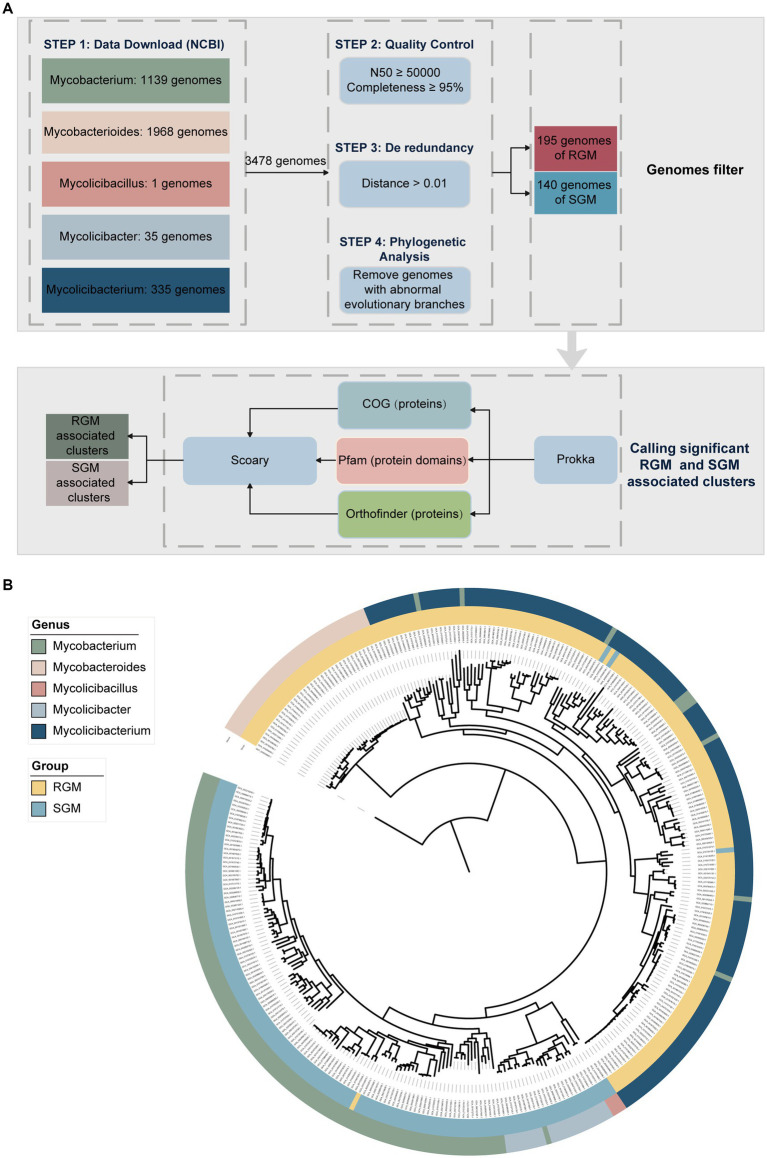
Research design and NTM genome dataset used in the analysis. **(A)** Overview of the methods used for calling RGM- and SGM-associated genes (proteins) and protein domains. Initially, a dataset of NTM genomes was established for comparative analysis. A strict quality control process was applied to remove low-quality or redundant genomes. Different approaches based on existing functional annotation (COG, Pfam, and orthology) were employed for clustering of gene-coding sequences in the NTM genomes. RGM- and SGM-associate gene (protein) and protein domain clusters were called through the Scoary comparative analysis. **(B)** Maximum likelihood phylogenetic tree of 335 high-quality and non-redundant NTM genomes based on the concatenated alignment of single-copy homogenous genes. The outer ring represents the genus, while the inner ring represents the growth rate of NTM.

In this study, a total of 3,478 NTM genome sequences (3,176 draft and 302 complete genomes) were obtained from the NCBI database (Figure 1A). After quality control and de-redundancy, we identified 335 high-quality non-redundant genomes covering 187 NTM species, with an N50 value of at least 50,000 and completeness of at least 95% (Supplementary Table S1). Among them, 195 genomes belonged to RGM and 140 genomes belonged to SGM.

To investigate the phylogeny of NTM, we identified 198 single-copy orthologous genes from the 335 genomes using OrthoFinder, and reconstructed a phylogenetic tree based on these genes with *Nocardia farcinica* IFM 10152 (GCA_000009805.1) as the outgroup (Figure 1B). The phylogenetic analysis revealed highly clonal distribution of rapid-growing NTM (RGM) and slow-growing NTM (SGM). RGM mainly belonged to the *Mycobacteroides* and *Mycolicibacterium* genera, whereas SGM mainly belonged to the *Mycobacterium*, *Mycolicibacter* and *Mycolicibacillus* genera, which highlights a strong correlation between taxonomy/phylogeny and growth rate in NTM.

In addition, we detected some misnamed NTM strains based on phylogenetic analysis. For instance, *Mycobacterium palauense* CECT 8779 (GCA_002592005.1), *Mycobacterium grossiae* DSM 104744 (GCA_008329645.1), *Mycobacterium neglectus* CECT 8778 (GCA_002591975.1), *Mycobacterium neumannii* CECT 8766 (GCA_002245615.1), *Mycobacterium lehmannii* CECT 8763 (GCA_002245535.1), *Mycobacterium lehmannii* IS-1744 (GCA_001499925.1), *Mycobacterium kyogaense* NCTC 11659 (GCA_003254575.1), *Mycobacterium aquaticum* RW6 (GCA_002086485.1), and *Mycobacterium syngnathidarum* 27,335 (GCA_001942625.1) were classified as *Mycobacterium*, but their phylogenetic analysis indicated that they should belong to the *Mycolicibacterium* genus. *Mycobacterium novum* JCM 6391 (GCA_010726505.1) was categorized as *Mycobacterium*, but its phylogenetic analysis indicated that it should belong to *Mycolicibacter*. Our findings were further validated by Average Nucleotide Identity (ANI) analysis, as the aforementioned genomes clustered consistently with the observed taxonomic groups (Supplementary Figure S2; Supplementary Table S8). Overall, the taxonomy of NTM species for these strains need to be re-evaluated.

We also observed that the growth rates of some strains were inconsistent with their phylogenetic positions. For example, M*ycolicibacterium tusciae* JS617 (GCA_000243415.3), *Mycolicibacterium tusciae* DSM 44338 (GCA_002086795.1), *Mycobacterium doricum* JCM 12405 (GCA_010728155.1), and *Mycobacterium bourgelatii* JCM 30725 (GCA_010723575.1) exhibited atypical growth phenotypes, which is consistent with previous findings confirming that these slow growing NTM species are located within the RGM lineage (Devulder et al., 2005; Mignard and Flandrois, 2008; Tortoli, 2012; Tortoli et al., 2017).

### Genome analysis revealed differential genomic features between RGM and SGM

**Figure 2:**
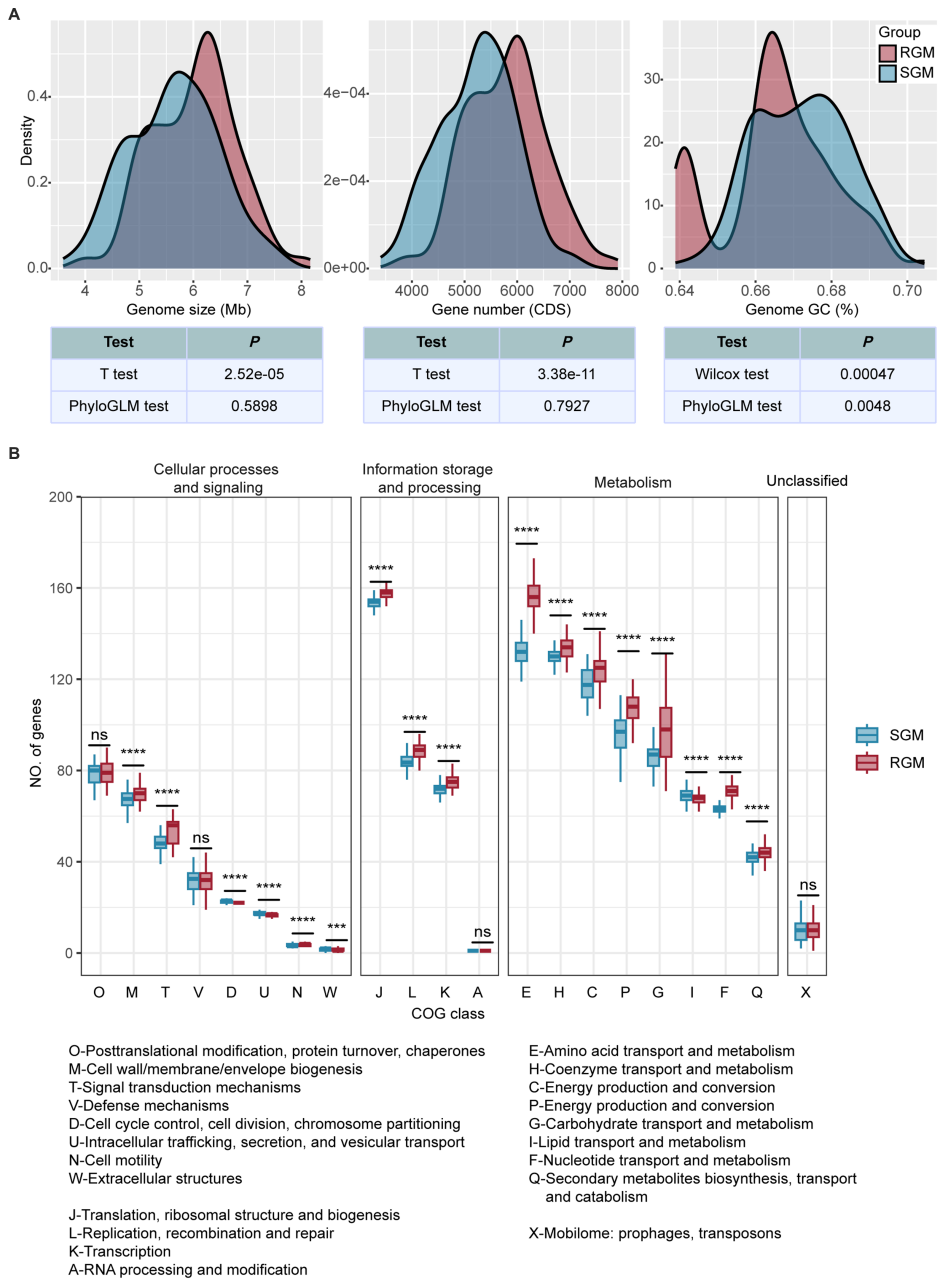
Differences in genomic features and gene categories (COG) between RGM and SGM genomes. **(A)** Density plots of genome size, gene number, and genome GC content. The color of the kernels represents growth rate (red represents RGM, and blue represents SGM). Methods used for the comparison between RGM and SGM genomes and their results are listed blow the density plots. **(B)** Differences of gene numbers in COG categories between RGM and SGM genomes. The color of box plots represent the growth rate. *P*-values were calculated from t-tests (‘*’ indicates *p* < 0.05, ‘**’ indicates *p* < 0.01, ‘***’ indicates *p* < 0.001, ‘****’ indicates *p* < 0.0001, and ‘ns’ idenoted as not significant). Please refer to Supplementary Table S2 for detailed meanings of COG category abbreviations.

Genome analysis revealed significant differences in genome size, gene number, and GC content between RGM and SGM (Figure 2A). RGM exhibited a larger genome size (6,015,739 bp for RGM: 5,635,503 bp for SGM, *p* < 0.001, *t*-test), a greater gene number (5,796 CDS for RGM: 5,241 CDS for SGM, *p* < 0.001, t-test), and a lower GC content (67.19% for SGM: 66.57% for RGM, *p* < 0.01, Wilcoxon test and PhyloGLM test) than SGM, which was in agreement with some previous research (Turenne, 2019). Incidentally, as a main environmental microorganism, the genomes of NTMs displayed substantial variation within RGM/SGM genomes for adaptation to various environments (Turenne, 2019).

We then used 21 COG categories (excluding the “Function unknown” category) to investigate RGM and SGM genomes using both t-test (Figure 2B) and PhyloGLM (Supplementary Figure S1). We found that 12 COG categories were significantly enriched in RGM, seven of which were related to metabolism; while only one category was significantly enriched in SGM (lipid transport and metabolism). Among the 12 categories enriched in RGM, such as “Amino acid transport and metabolism (E)” and “Transcription (K),” which have been reported to be necessary for rapid growth of RGM (Bachmann et al., 2019). On the other hand, the lipid transport and metabolism (I) category was significantly enriched in SGM, which has been reported to improve the environmental adaptability of SGM (Tran et al., 2019; Pereira and Ramos, 2020).

Overall, these differences reflect genome adaptations in responsible for differential growth rates between RGM and SGM. RGM has a larger genome size, a greater gene number, and stronger metabolism for adaptation of their rapid growth requirements; SGM has a higher GC content and stronger lipid transport and metabolism in order to enhance their genome stability and environmental adaptability for slower growth.

### Identification of rapid- and slow-growing featured genes In RGM and SGM

To identify featured genes enriched in the RGM and SGM genomes, we annotated all putative protein-coding sequences using COG and employed Scoary to determine whether the COG genes (genes mapped to the same COG ID) were significantly associated with RGM or SGM (Levy et al., 2018). The genes with significant enrichment in RGM and SGM were considered as RGM or SGM featured/associated genes.

**Figure 3:**
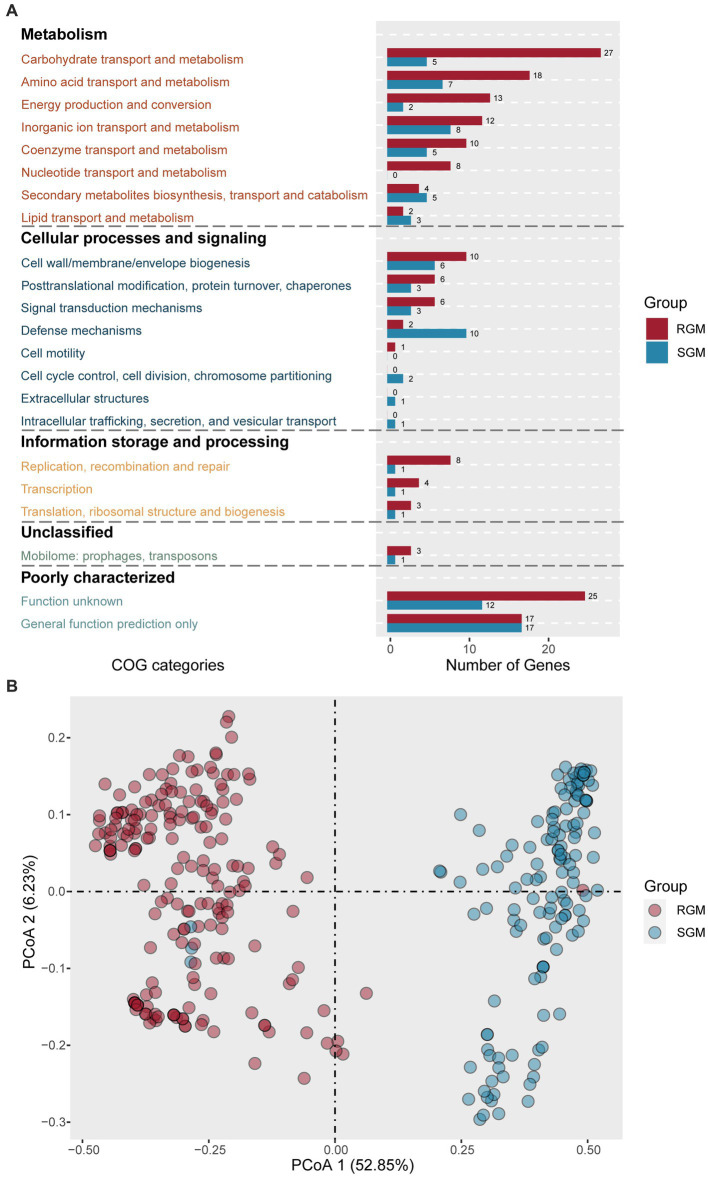
Identification of genes (proteins) associated with RGM and SGM based on the COG database. **(A)** Number of RGM- and SGM- associated genes (proteins) in COG categories. The color of the bar plot represents the growth rate. **(B)** PCoA of RGM and SGM genomes based on the top 20 most significant differential genes (proteins) associated with RGM and SGM. The color of the dots represents the growth rate.

Using the above-described methodology, 170 RGM-associated genes and 87 SGM-associated genes were predicted (Supplementary Table S3), most of which were associated with metabolism, followed by cellular processes, signaling, and information storage and processing (Figure 3A). Comparative analysis unveiled that RGM had more enriched genes in metabolism and transport of essential substances (such as carbohydrates, amino acids, nucleotides, ions, and coenzymes), and energy production and transport.

First, metabolism related genes rank first in both RGM-associated genes and SGM-associated genes. Among the eight metabolism-related categories, RGM had more significantly enriched genes in six ones, with five of the six categories displaying an increase of at least 50% compared to SGM (Carbohydrate transport and metabolism, Amino acid transport and metabolism, Energy production and conversion, Nucleotide transport and metabolism, Coenzyme transport and metabolism). Notably, RGM owned eight enriched genes in “nucleotide transport and metabolism” category (encoding PucL, AlC, SsnA, FuI1, PurU, GuaA1, UdK, and AllE) but no such gene was detected in SGM, as it is known that RGM needs a stronger nucleotide metabolism for facilitating its fast growth and reproduction (Peebo et al., 2015). Specically, PucL, AlC, SsnA, and FuI1 were involved in uric acid metabolism, PurU and GuaA1 were linked to purine synthesis, UdK was associated with pyrimidine synthesis, and AllE was related to urea metabolism. On the other hand, SGM had more enriched genes in “Secondary metabolites biosynthesis, transport and catabolism” and “Lipid transport and metabolism” categories, which have been reported in favor of slow-growth of bacteria (Shin et al., 2015; Bouam et al., 2018).

As for cellular processes and signaling, RGM had more enriched genes related to synthesis of cell walls and membranes, post-translational modification, signal transduction, and cell motility; SGM had more enriched genes related to “Defense mechanisms,” “Cell cycle control, cell division, chromosome partitioning” and “Intracellular trafficking, secretion, and vesicular transport” and “Extracellular structures.” Importantly, more defense mechanism associated genes (encoding PhD, StbD, and LprI) were detected in SGM such as toxin-antitoxin components, which have been reported to facilitate bacterial survival under harsh conditions (Eroshenko et al., 2020).

Concerning information storage and processing, RGM had more enriched genes related to “Replication, recombination and repair,” “Transcription,” and “Translation, ribosomal structure and biogenesis,” which were fit for their rapid growth as previously reported (Peebo et al., 2015; Bachmann et al., 2019). On the other hand, we noticed that SGM had three enriched genes (encoding UdG, PutA1, and ThpR), which had been reported to play roles in maintaining DNA stability for slow growth (Peebo et al., 2015; Lupoli et al., 2016).

Overall, due to their differential survival states, RGM had more featured genes associated with “Carbohydrate transport and metabolism,” “Amino acid transport and metabolism,” “Energy production and conversion,” “Nucleotide transport and metabolism,” “Replication, recombination and repair,” “Transcription,” and “Translation, ribosomal structure and biogenesis”(RGM/SGM > 2), whereas SGM had more featured genes contributing to “Defense mechanisms” (SGM/RGM > 2).

We then focused on the top 20 significantly enriched genes in RGM (Table 1) and SGM (Table 2), most of which were related to metabolism. Specifically, RGM and SGM had 13 and 12 distinct metabolism-related featured genes, respectively. The 13 RGM featured metabolism-related genes were classified into “Amino acid transport and metabolism” (4 genes), “Nucleotide transport and metabolism” (3 genes), “Energy production and conversion” (2 genes), “Secondary metabolites biosynthesis, transport and catabolism” (2 genes), “Carbohydrate transport and metabolism” (1 gene), and “Inorganic ion transport and metabolism” (1 gene). Meanwhile, the 12 metabolism-related genes in SGM were associated with “Amino acid transport and metabolism” (4 genes), “Carbohydrate transport and metabolism” (2 genes), “Secondary metabolites biosynthesis, transport and catabolism” (2 genes), “Inorganic ion transport and metabolism” (2 genes), “Lipid transport and metabolism” (1 gene), and “Energy production and conversion” (1 gene).

**Table 1:** Function and categorization of top 20 feature genes in RGM.

COG accession	Odds ratio	BH adjusted p	COG symbol	COG description	COG class	COG class summary
COG3842	274.125	3.38E-66	PotA	ABC-type Fe3^+^/spermidine/putrescine transport systems, ATPase component	E	Metabolism
COG1279	184.594	1.16E-61	ArgO	Arginine exporter protein ArgO	E	Metabolism
COG3195	143.130	1.28E-58	PucL	2-oxo-4-hydroxy-4-carboxy--5-ureidoimidazoline (OHCU) decarboxylase (uric acid degradation)	F	Metabolism
COG3733	144.375	3.58E-58	TynA	Cu2^+^-containing amine oxidase	Q	Metabolism
COG4521	inf	1.59E-53	TauA	ABC-type taurine transport system, periplasmic component	P	Metabolism
COG4126	63.8180	3.19E-46	DcG1	Asp/Glu/hydantoin racemase	E	Metabolism
COG4292	58.667	5.31E-46	LtrA	Low temperature requirement protein LtrA (function unknown)	S	Poorly characterized
COG1953	61.559	1.38E-45	FuI1	Cytosine/uracil/thiamine/allantoin permease	FH	Metabolism | Metabolism
COG2350	42.618	1.68E-41	YciI	YciI superfamily enzyme, includes 5-CHQ dehydrochlorinase, contains active-site pHis	QR	Metabolism | Poorly characterized
COG0518	66.5448	2.46E-41	GuaA1	GMP synthase, glutamine amidotransferase domain	F	Metabolism
COG1188	37.9838	8.80E-41	HslR	Ribosomal 50S subunit-recycling heat shock protein, contains S4 domain	J	Information storage and processing
COG2954	inf	1.06E-34	CytH	CYTH domain, found in class IV adenylate cyclase and various triphosphatases	R	Poorly characterized
COG4913	26.25	5.70E-34		Uncharacterized conserved protein, contains a C-terminal ATPase domain	S	Poorly characterized
COG4681	32.268	2.55E-33	YaeQ	Uncharacterized conserved protein YaeQ, suppresses RfaH defect	S	Poorly characterized
COG1139	inf	4.48E-32	LutB	L-lactate utilization protein LutB, contains a ferredoxin-type domain	C	Metabolism
COG2723	inf	1.14E-31	BglB	Beta-glucosidase/6-phospho-beta-glucosidase/beta-galactosidase	G	Metabolism
COG1135	inf	7.63E-31	AbcC	ABC-type methionine transport system, ATPase component	E	Metabolism
COG0830	43.246	2.33E-30	UreF	Urease accessory protein UreF	O	Cellular processes and signaling
COG1556	inf	5.86E-30	LutC	L-lactate utilization protein LutC, contains LUD domain	C	Metabolism
COG1742	26.613	8.68E-30	YnfA	Uncharacterized inner membrane protein YnfA, drug/metabolite transporter superfamily	R	Poorly characterized

Odds ratios and *p*-values were calculated using Scaory.

**Table 2:** Function and categorization of top 20 feature genes in SGM.

COG accession	Odds ratio	BH adjusted p	COG symbol	COG description	COG class	COG class summary
COG1168	0.0027	2.90E-69	MalY	Bifunctional PLP-dependent enzyme with beta-cystathionase and maltose regulon repressor activities	ER	Metabolism | Poorly characterized
COG1045	0.018	4.15E-46	CysE	Serine acetyltransferase	E	Metabolism
COG2815	0	3.61E-40	PASTA	PASTA domain, binds beta-lactams	M	Cellular processes and signaling
COG4101	0.035	2.09E-32	RmlC	Uncharacterized conserved protein, RmlC-like cupin domain	R	Poorly characterized
COG2919	0.0497	6.57E-31	FtsB	Cell division protein FtsB	D	Cellular processes and signaling
COG0538	0.041	2.56E-30	IcD	Isocitrate dehydrogenase	C	Metabolism
COG3848	0.057	1.86E-25	PykA2	Phosphohistidine swiveling domain of PEP-utilizing enzymes	T	Cellular processes and signaling
COG2899	0.051	6.62E-25	TerC2	Uncharacterized TerC-related membrane protein, DUF475 domain	P	Metabolism
COG1085	0.082	2.21E-21	GalT	Galactose-1-phosphate uridylyltransferase	G	Metabolism
COG3360	0.056	2.66E-21		Flavin-binding protein dodecin	R	Poorly characterized
COG1982	0.010	1.68E-20	LdcC	Arginine/lysine/ornithine decarboxylase	E	Metabolism
COG2046	0	9.30E-20	MeT3	ATP sulfurylase (sulfate adenylyltransferase)	P	Metabolism
COG0439	0.109	4.88E-19	AccC	Biotin carboxylase	I	Metabolism
COG2273	0.114	1.42E-18	BglS	Beta-glucanase, GH16 family	G	Metabolism
COG0706	0.089	1.96E-18	YidC	Membrane protein insertase Oxa1/YidC/SpoIIIJ	M	Cellular processes and signaling
COG3485	0.102	3.44E-18	PcaH	Protocatechuate 3,4-dioxygenase beta subunit	Q	Metabolism
COG1514	0.095	3.19E-16	ThpR	RNA 2′,3′-cyclic phosphodiesterase (2′-5’ RNA ligase)	J	Information storage and processing
COG1647	0.105	7.28E-16	YvaK	Esterase/lipase	Q	Metabolism
COG4129	0.0665	1.68E-15	YgaE	Uncharacterized membrane protein YgaE, UPF0421/DUF939 family	S	Poorly characterized
COG0709	0.1295	3.39E-15	SelD	Selenophosphate synthase	E	Metabolism

Odds ratios and *p*-values were calculated using Scaory.

Among the top20 genes related to “Amino acid transport and metabolism,” RGM are closely associated with amino acid transport processes, while SGM owns more diverse amino acid metabolic pathways, with the involved proteins and their functions playing significant roles in environmental adaptation and oxidative stress response. Among the four amino acid metabolic pathways related to RGM, three gene encoded proteins closely associated with amino acid transport: PotA (COG3842, top-1), ArgO (COG1279, top-2), and AbcC (COG1135, top-17) are the members of the ABC-type transport systems, responsible for transporting polyamines, arginine, lysine, and ornithine. Additionally, Dcg1 (COG4126, top-6) catalyzes the stereoisomerization of amino acids/compounds like Asp/Glu/hydantoin, however, the specific cellular function of Dcg1 still requires further exploration. On the other hand, SGM owns some proteins like MalY (COG1168, top-1), CysE (COG1045, top-2), and LdcC (COG1982, top-11) in amino acids metabolism pathways (such as serine, arginine/lysine/ornithine, and beta-cysteine). Additionally, a selenium-metabolism related protein, SelD (COG0709, top-20), can enhance SGM’s oxidative stress response, aiding the bacteria in better adapting to their environment.

Furthermore, RGM and SGM exhibit notable differences in “Carbohydrate transport and metabolism” and “Energy production and conversion.” RGM demonstrates a more diverse substrate utilization capacity. The top-20 genes associated with RGM included two genes associated with L-lactate metabolism (COG1139 encoding LutB, top-15; COG1556 encoding LutC, top-19), and one gene involved in galactose and glucose metabolism (COG2723 encoding BglB, top-16). In SGM, two carbohydrate metabolism-related genes are enriched, such as COG1085 (encoding GalT, top-9) and COG2273 (encoding BglS, top-14), which are associated with galactose and glucose metabolism, as well as one energy metabolism-related gene encoding IcD (COG0538, top-6), participating in the tricarboxylic acid (TCA) cycle. The differential expression of these genes reflects the distinct variations in energy utilization and environmental adaptation between RGM and SGM. Additionally, three genes related to nucleotide metabolism (COG3195 encoding PucL, top-3; COG1953 encoding FuI1, top-8; COG0518 encoding GuaA1, top-10) and one gene related to ribosome structure were found (COG1188, top-11). Within “Secondary metabolites biosynthesis, transport and catabolism,” RGM-associated TynA (COG3733, top-4) tends to utilize amines and SGM-associated YvaK (COG1647, top-18) tends to utilize esters.

Notably, five genes encoding transport proteins were found among the top 20 genes associated with RGM, facilitating the transport of nucleotides, ions, amino acids, and sugars, including COG3842 (top-1), COG1279 (top-2), COG4521 (top-5), COG1953 (top-8), and COG1135 (top-17). On the orther hand, there are two genes (COG2815: top-3, COG0706: top-15) involved in Cell wall/membrane/envelope biogenesis in SGM. Research has indicated that the synthesis of cell membrane consumes a significant amount of energy, which may asscioate to slow growth of SGM (Pereira and Ramos, 2020). These differential enrichment of metabolism-related featured genes indicated its important and even decisive role in distinct growth rates between RGM and SGM.

Based on the top 20 genes in RGM and SGM, a PCoA analysis was conducted using the 335 RGM and SGM genomes (Figure 3B). The analysis revealed that the 40 genes could significantly differentiate RGM from SGM, indicating the credibility of our featured gene screening/classification strategy.

### Identification of rapid- and slow-growing associated/featured protein domains in RGM and SGM

Protein domains are typically highly conserved across different species, which play important roles in protein function/structure (Levy et al., 2018). Similar to the identification of RGM- and SGM-associated genes, the identification of RGM- and SGM-associated protein domains involves two steps: protein domain annotation using PfamScan, followed by the identification of RGM- and SGM-associated protein domains using Scoary (Supplementary Table S4). To further explore the function of identified RGM- and SGM-associated protein domains, we performed COG annotations corresponding to those protein domains (Supplementary Table S5).

**Figure 4:**
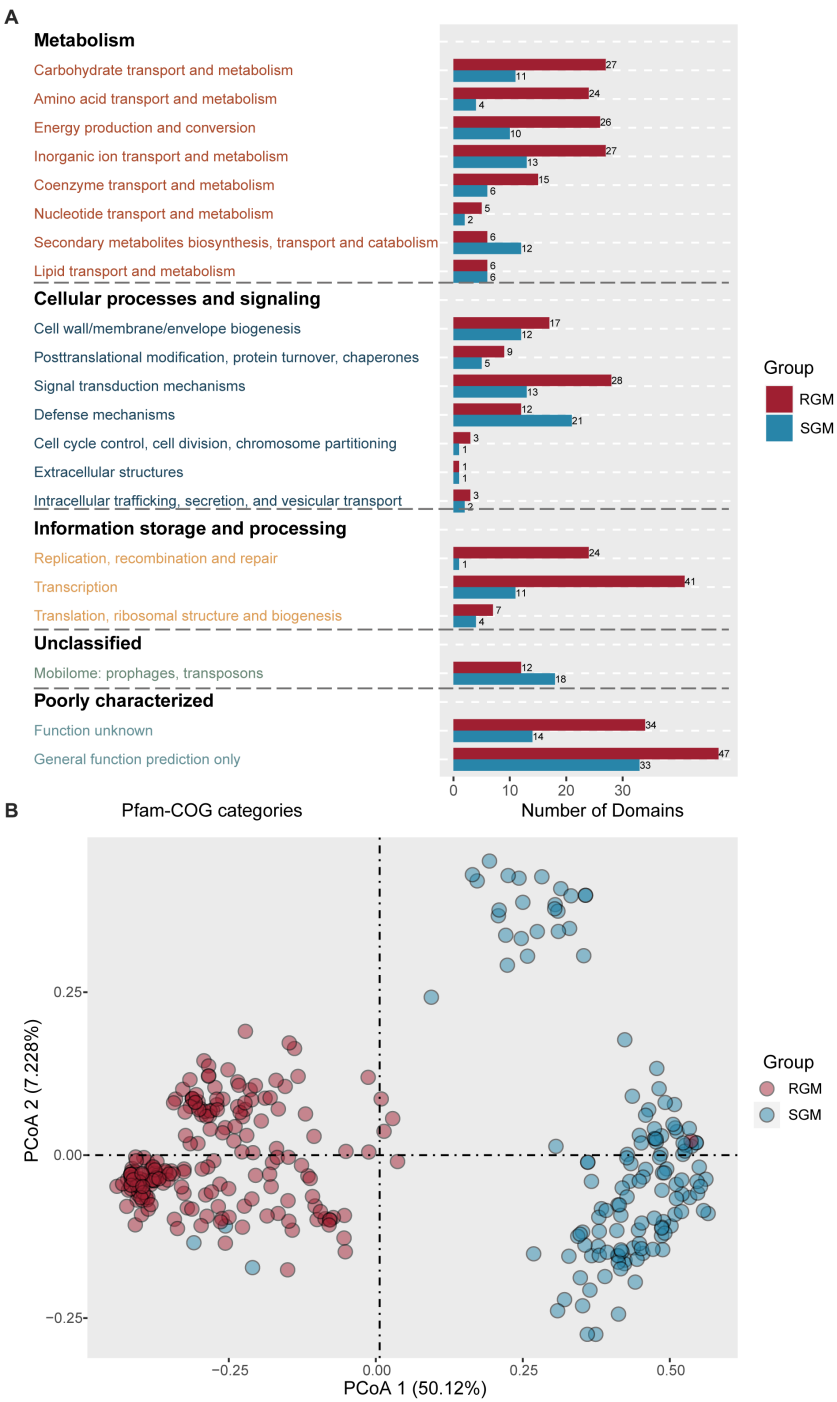
Identification of protein domains associated with RGM and SGM based on the PFam database. **(A)** Number of RGM- and SGM- associated protein domains in COG categories. The color of the bar plot represents the growth rate. **(B)** PCoA of RGM and SGM genomes based on the top 20 most significant differential protein domains associated with RGM and SGM. The color of the dots represents the growth rate.

275 RGM-associated protein domains and 144 SGM-associated protein domains were predicted (Supplementary Table S4). Metabolism related protein domains rank first in both RGM- and SGM-associated protein do mains (Figure 4A). Among the eight metabolism-related categories, RGM owned much more significantly enriched protein domains in six of them, all of which displayed an increase of at least 50% compared to SGM (Carbohydrate transport and metabolism, Amino acid transport and metabolism, Energy production and conversion, Inorganic ion transport and metabolism, Nucleotide transport and metabolism, Coenzyme transport and metabolism). Importantly, COG annotation of these enriched protein domains in RGM and SGM (Figure 4A) showed similar results to that of the enriched proteins (Figure 3A), indicating more active metabolism of some main substances (carbohydrate, amino acid, inorganic ion, nucleotide, coenzyme) and energy (energy production and conversion) for adapt to rapid growth in RGM. On the other hand, SGM had much more enriched protein domains related to “Secondary metabolites biosynthesis, transport and catabolism” than RGM (12/6); SGM had identical enriched protein domains in “Lipid transport and metabolism” category to RGM. Secondary and lipid metabolisms have been reported to play important roles in slow growing bacteria (Peebo et al., 2015; Lupoli et al., 2016), which is also consistent with COG annotation of enriched proteins (Figure 3A).

As for cellular processes and signaling, RGM had more enriched protein domains in “Signal transduction mechanisms” (RGM/SGM > 2: 28/13) categories, while SGM had much more enriched protein domains in “Defense mechanisms” category (SGM/RGM: 21/13) for adapt to bacterial slow growth. The above findings are consistent with COG annotation of enriched proteins (Figure 3A). We then focused on the 21 enriched defense-mechanism related protein domains in SGM. Most of them (12/21) belonged to toxin-antitoxin systems, which was also in agreement with COG annotation of enriched proteins (Figure 3A). Interestingly, in the 21 defense-mechanism related protein domains, we also observed four protein domains enriched in CRISPR-Cas system (PF09827: CRISPR associated protein Cas2; PF13518: Helix-turn-helix domain; PF13683: Integrase core domain; and PF13565: Homeodomain-like domain), which have been documented to avoid the invasion of foreign nucleic acids from virus/vector for adapting to hostile environments in SGM (Makarova et al., 2011).

Concerning information storage and processing, RGM had more protein domains in “Replication, recombination and repair,” “Transcription,” and “Translation, ribosomal structure and biogenesis” categories than SGM, again confirming the conclusions from the above protein COG annotations (Figure 3A).

We then analyzed the top 20 significantly enriched protein domains in RGM (Table 3), among which five corresponded to seven proteins from the top 20 RGM enriched proteins (Table 1). PF08402 (TOBE domain, top-5) corresponds to MalK and PotA proteins, as ABC transporter proteins for transporting ions, amino acids, and sugars into cell (Kashiwagi et al., 1993). PF02589 (LuD domain, top-9) is associated with the LutB and LutC proteins for lactate metabolism (Hwang et al., 2013). PF00232 (Glycosyl hydrolase family 1, top-10) is related to BglB protein for glucose and galactose metabolism (Wright et al., 1992). In addition, we also found that two branched-chain amino acid transport protein domains, PF05437 (top-16) and PF03591 (top-17) corresponded to two enriched branched-chain amino acid transport protein AzlD and AzlC, respectively, which had been reported to play important roles in rapid growth of RGM (Bachmann et al., 2019).

**Table 3:** Top 20 Feature protein domains in RGM.

Pfam accession	Odds ratio	BH adjusted p	Domain symbol	Domain description
PF02698	269.278	3.37E-66	DUF218	DUF218 domain
PF02557	132.458	1.61E-55	VanY	D-alanyl-D-alanine carboxypeptidase
PF04328	69.048	2.42E-49	Sel_put	Selenoprotein, putative
PF19460	42.1498	4.08E-42	DUF5997	Family of unknown function (DUF5997)
PF08402	36.816	7.04E-39	TOBE_2	TOBE domain
PF03334.	inf	8.41E-37	PhaG_MnhG_YufB	Na^+^/H^+^ antiporter subunit
PF09972	32.667	2.26E-35	DUF2207	Predicted membrane protein (DUF2207)
PF07152	34.8307	7.50E-35	YaeQ	YaeQ protein
PF02589	inf	3.82E-32	LUD_dom	LUD domain
PF00232	inf	1.51E-31	Glyco_hydro_1	Glycosyl hydrolase family 1
PF13727	20.640	2.46E-31	CoA_binding_3	CoA-binding domain
PF13835	21.614	8.43E-30	DUF4194	Domain of unknown function (DUF4194)
PF02733	inf	2.40E-29	Dak1	Dak1 domain
PF13796	162.167	2.60E-29	Sensor	Putative sensor
PF11855	20.896	2.90E-29	DUF3375	Protein of unknown function (DUF3375)
PF05437	inf	3.08E-29	AzlD	Branched-chain amino acid transport protein (AzlD)
PF03591	inf	3.08E-29	AzlC	AzlC protein
PF09278	40.795	4.22E-29	MerR-DNA-bind	MerR, DNA binding
PF13555	19.333	1.19E-28	AAA_29	P-loop containing region of AAA domain
PF17885	inf	1.88E-28	Smoa_sbd	Styrene monooxygenase A putative substrate binding domain

Odds ratios and *p*-values were calculated using Scaory.

On the other hand, among the 20 most significantly enriched protein domains in SGM (Table 4), we detected five protein domains related to defense mechanism for environmental adaptation of SGM. PF09957 (top-3) is associated with a bacterial antitoxin of type II TA system VapB6 in toxin-antitoxin system. PF12051 (top-5) corresponds to two ABC-2-transporter-like clan transporters YadH (ABC-type multidrug transport system, permease component) and NatB (ABC-type Na^+^ efflux pump, permease component NatB) in responsible for antibiotic and Na^+^ ion cellar exclusion. PF01436 (NHL repeat, top-8) corresponds to a defense mechanism related protein VgB (streptogramin lyase). PF07311 (Dodecin domain, top-16) is associated with the flavin-binding protein dodecin for storing riboflavin or resisting free radicals/oxygen stress (Bieger et al., 2003). It worth noting that PF01747 (ATP-sulfurylase domain, top-19) and PF14306 (PUA-like domain, top-20) belong to the ATP sulfurylase protein, which have been reported to participate in sulfate oxidative metabolism for bacterial survival in host and pathogenicity (Hudson and York, 2012).

**Table 4:** Top 20 Feature protein domains in SGM.

Pfam accession	Odds ratio	BH adjusted p	Domain symbol	Domain description
PF10921	0.008	1.96E-56	DUF2710	Protein of unknown function (DUF2710)
PF10904	0.010	3.44E-48	DUF2694	Protein of unknown function (DUF2694)
PF09957	0.026	1.61E-40	VapB_antitoxin	Bacterial antitoxin of type II TA system, VapB
PF10817	0.028	1.09E-38	DUF2563	Protein of unknown function (DUF2563)
PF12051	0.032	2.10E-37	DUF3533	Protein of unknown function (DUF3533)
PF07098	0.028	5.85E-37	DUF1360	Protein of unknown function (DUF1360)
PF04185	0.038	7.49E-34	Phosphoesterase	Phosphoesterase family
PF01436	0.0338	5.99E-32	NHL	NHL repeat
PF14114	0.0628	9.87E-27	DUF4286	Domain of unknown function (DUF4286)
PF04332	0.0458	3.24E-26	DUF475	Protein of unknown function (DUF475)
PF13618	0.0748	2.03E-23	Gluconate_2-dh3	Gluconate 2-dehydrogenase subunit 3
PF14362	0.090	5.99E-22	DUF4407	Domain of unknown function (DUF4407)
PF01087	0.082	2.65E-21	GalP_UDP_transf	Galactose-1-phosphate uridyl transferase, N-terminal domain
PF02744	0.082	2.65E-21	GalP_UDP_tr_C	Galactose-1-phosphate uridyl transferase, C-terminal domain
PF07311	0.056	3.18E-21	Dodecin	Dodecin
PF13529	0.090	2.92E-20	Peptidase_C39_2	Peptidase_C39 like family
PF19812	0.057	5.52E-20	DUF6295	Family of unknown function (DUF6295)
PF12391	0.102	6.21E-20	PCDO_beta_N	Protocatechuate 3,4-dioxygenase beta subunit N terminal
PF01747	0	1.07E-19	ATP-sulfurylase	ATP-sulfurylase
PF14306	0	1.07E-19	PUA_2	PUA-like domain

Odds ratios and p-values were calculated using Scaory.

Similarly, PCoA analysis of the abovementioned 40 protein domains showed that RGM and SGM strains could be significantly distinguished into two clusters, indicating high correlation between these protein domains and bacterial growth rates (Figure 4B).

### Identification of co-occured orthogroups associated with RGM and SGM

We only annotated ~10% of genes/protein domains using COG and Pfam, since most NTM’s genome were poorly characterized in COG. To explore the gene functions without accurate annotations, OrthoFinder was utilized to cluster all putative protein-coding sequences of the entire genome into orthogroups based on homology (Emms and Kelly, 2015), followed by Scoary for identify the orthogroups with rapid and slow growth rates (Supplementary Table S6). The spearman correlation analysis of classified orthogroups revealed co-occured ortholog gene clusters. As a result, the co-occurion of these gene clusters suggests their similar roles in function, providing valuable insights into the NTM’s genome.

**Figure 5:**
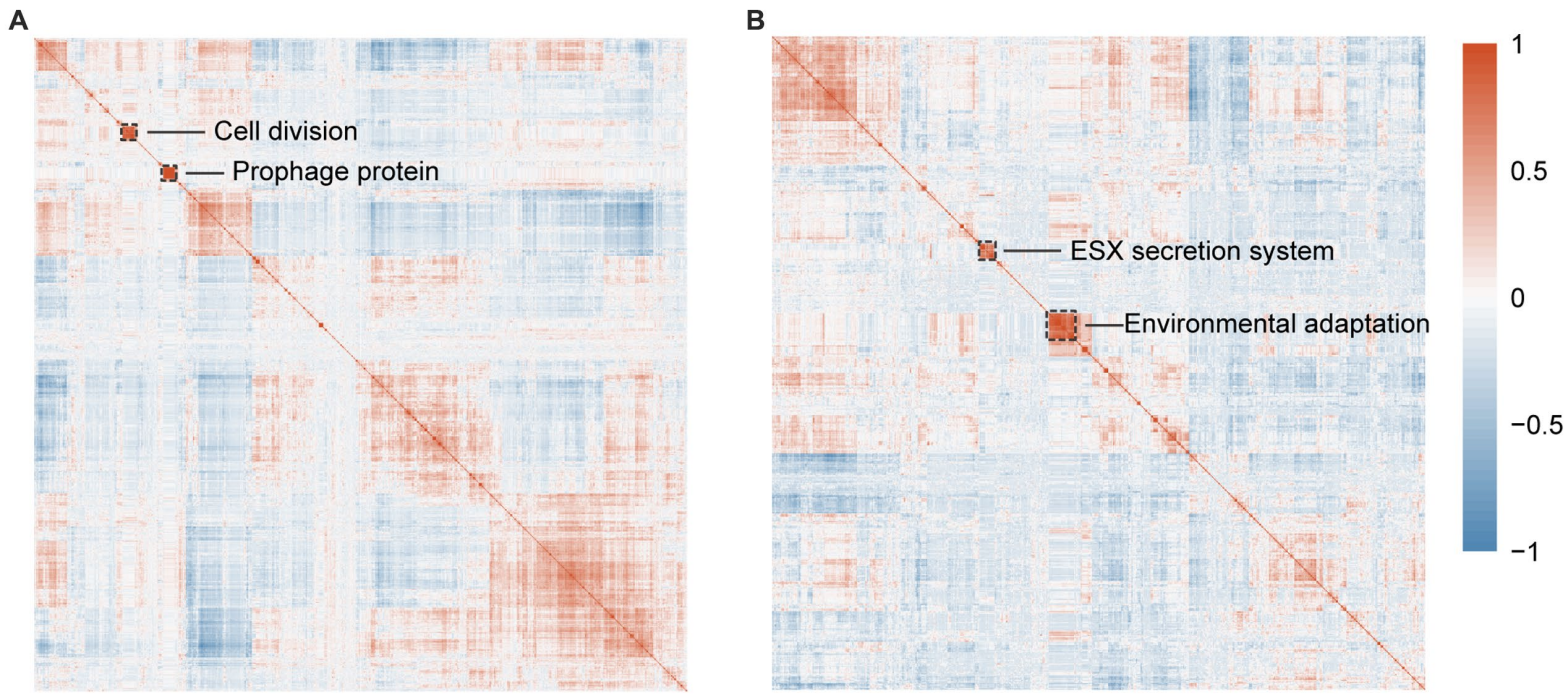
Identification of orthogroups associated with RGM and SGM using OrthoFinder. **(A)** Correlation heatmap with hierarchical clustering of 1,316 significantly enriched RGM-associated orthogroups predicted by Scoary. **(B)** Correlation heatmap with hierarchical clustering of 891 significantly enriched SGM-associated orthogroups predicted by Scoary. The dashed boxes highlight orthogroups with the most significant correlations.

As for RGM related co-occured orthogroups, we mainly focused on two co-occured gene clusters with bacterial division and bacteriophage functions (Figure 5A), indicating the important roles of these two functions in bacterial rapid growth. In the first cluster, five orthogroups are associated with bacterial division, suggesting that the other 17 poorly characterized genes in the cluster have similar function. Specifically, OG0007945 (PF00004, PF01580, PF17866), OG0009308 (PF00004, PF01580, PF17866), and OG0009980 encode a DNA segregation ATPase FtsK, and OG0009980 encodes a integrase/recombinase XerD, all of which have been reported to facilitate cell division (Iyer et al., 2004). OG0009140 encodes the DnaJ protein that is responsible for the activation of DnaK, which play important roles in degrade and fold defective proteins in cell division (Lupoli et al., 2016). In the second cluster of co-occured genes, a significant proportion of proteins (22/27) encode for the tail protein of bacteriophages, indicating more horizontal gene transfer events in RGM (Rathnapala et al., 2023). Following PHASTER annotation (Supplementary Table S9), the annotation results of these single-copy homologous genes indicate their predominant affiliation with prophage genes. These prophages have been identified in the genus *Mycobacterium*, aligning coherently with the COG annotations. For example, when considering the gene most prominently present in the Orthogroup within the second cluster, encoded by the GCA_017189435.1 genome, a parallel linkage becomes evident between the bacteriophage genes annotated by COG and those identified through PHASTER (Supplementary Figure S3). This observation proves the consistency between COG and PHASTER annotations in identifying bacteriophage genes. As a result, horizontal gene transfer could facilitate new gene acquisition for metabolism and rapid growth (Rathnapala et al., 2023).

As for SGM related co-occured orthogroups, we mainly focused on two co-occured gene clusters with bacterial secretion and environmental adaptation functions (Figure 5B). The first cluster is predicted to be associated with bacterial secretion system and virulence/pathogenicity. Specifically, OG0008539 encodes an ESX-3 secretion system protein EccE3, and OG0010180 (PF11203) encodes a putative type VII ESX secretion system protein translocon, all of which have been reported to be related to bacterial virulence/pathogenicity (Thakur et al., 2019). The second cluster is predicted to be associated with environmental adaptation. Specifically, OG0009821 (COG0515, PF00069) encodes a serine/threonine protein kinase that plays roles in bacterial signal transduction in response to various environments (Prisic and Husson, 2014). OG0010112 (COG1146, PF13187, PF14697) encodes H^+^-Na^+^-translocating ferredoxin for adjusting to specific environments by producing transmembrane electrochemical potentials (Vitt et al., 2022). OG0011183 (COG0318, PF00501, PF13193) encodes a fatty-acyl-CoA synthase in responsible for lipid metabolism and energy supply in SGM (Hisanaga et al., 2004).

## Discussion

In this study, we screened 335 high-quality, non-redundant NTM genome sequences covering 187 species from 3,478 online NTM genomes (as of January 2022), and then performed a comprehensive comparative genomic analysis to identify differential genomic characteristics and featured genes/protein domains in RGM and SGM. Our findings reveal that RGM has a larger genome size, more genes, lower GC content, and more featured genes/protein domains in metabolism of some main substances (e.g., carbohydrates, amino acids, nucleotides, ions, and coenzymes), energy metabolism, signal transduction, replication, transcription, and translation processes, which are essential for its rapid growth requirements. On the other hand, SGM has a smaller genome size, fewer genes, higher GC content, and more featured genes/protein domains in lipid metabolism and cellular defense mechanisms, which help enhance its genome stability and environmental adaptability for slower growth.

The rapid growth of RGM is primarily attributed to their more efficient and faster metabolism. Compared to SGM, RGM has more featured genes/protein domains associated with the metabolism of most main nutrient substances, such as carbohydrates, amino acids, nucleotides, ions, and coenzymes (Figure 3A; Figure 4A). For genomic characteristics, the higher AT content in RGM’s genome (Figure 2A) is positively associated with its efficiency in generating ATP for energy supply, which has been reported to be beneficial to the faster growth of RGM (Wu et al., 2012). In addition, our orthogroup analysis showed the enrichment of co-occured phage genes in RGM (Figure 5A). Phage genes are widely present in bacterial genomes and indicative of horizontal gene transfer (Rathnapala et al., 2023). Therefore, this enrichment can facilitate the acquisition of additional genes for accelerating metabolism and growth rates *via* horizontal gene transfer in RGM, which is also reflected in their larger genomes (Figure 2A). Incidentally, the comparatively weak defense mechanisms in RGM (Figure 3A; Figure 4A) makes them more prone to horizontal gene transfer and enlarge their genomic size.

Overall, the genomic characteristics and featured genes/protein domains in RGM (higher AT content, larger genome size, and more efficient metabolisms of main nutrient substances and phage related genes) reflect adaptive evolution and growth-rate selective advantages of RGM on a larger scale (Wang et al., 2010).

Currently, it is widely believed in the academic community that SGM have undergone a large-scale gene loss during their long evolutionary journey to adapt to harsher environments (Stinear et al., 2007; Wee et al., 2017; Tan et al., 2020). However, what genes they lost is not fully understood by current research. RGM, as the closest ancestor of SGM (differentiated earlier and with a larger genome) (Turenne, 2019), can more comprehensively reflect the genetic characteristics of their common ancestor and undoubtedly provide a good comparison object for detecting these genes lost at ancient evolutionary nodes. This study comprehensively revealed the genes lost in SGM during its long evolutionary journey to adapt to harsher environments through large-scale comparative genomics studies of RGM and SGM (including some metabolism related genes for most main nutrient substances), which might limit the utilization of these nutrient substances and further lowered the growth rate and smaller genome size in SGM.

On the other hand, although the growth rate of SGM is slower compared to RGM, they exhibit higher environmental adaptability and pathogenicity. They can survive in oligotrophic and hypoxic environments, some of which are parasitic/ symbiotic and can infect people, mostly human lung tissue, and further lead to various respiratory diseases and even death in severe cases (Pereira and Ramos, 2020). First, as for genomic characteristics, the high GC content in SGM has been reported to be usually positively correlated with environmental adaptability (Bentley and Parkhill, 2004; Mann and Chen, 2010; Šmarda et al., 2014). When exposed to harsh environments, genomic stability is usually improved by increasing genomic GC content (Šmarda and others 2014). In addition, our results indicated more genes involved in lipid and secondary metabolite metabolisms in SGM, which have been reported to be associated with energy storage and environmental adaptation (Tran et al., 2019; Pereira and Ramos, 2020).

Our research has also shown more genes related to cellular defense mechanisms in SGM, including toxin-antitoxin and ESX systems (Figure 3A; Figure 4A), which have been reported to enable bacteria to survive better in challenging environments within host organisms (Eroshenko et al., 2020). Specifically, the toxin-antitoxin system has been reported to be a significant contributor to bacterial dormancy and persistence (Eroshenko et al., 2020). The ESX secretion system, as one of the significant virulence factors in NTM, has been reported to play important roles in persistent infection and host immune evasion for adapting harsh environments (Fedrizzi et al., 2017; Thakur et al., 2019).

In conclusion, we investigated genomic differences in NTMs with different growth rates to explore the relationship between growth rate and underlying genetic mechanisms. Our results suggest that RGM utilize multiple sources of nutrition such as carbohydrates, amino acids and nucleotides, achieving rapid proliferation by increasing the type and number of these genes related to metabolism and signal transduction, replication, transcription, and translation processes. In contrast, SGM show the potential to adapt to harsh environments by utilizing/storing energy through lipid and secondary metabolites metabolisms. They have more cellular defense-related genes (toxin-antitoxin and ESX systemin) that facilitate their defense against external threats and enhance their pathogenicity in the host. The study has revealed featured genes and proteins of RGM and SGM. These findings could potentially offer new avenues for rapid identification of pathogens in clinical settings, thus providing strong support for diagnostic and therapeutic strategies. Our study highlights the role of identifying featured gene lost or acquisition during evolution in driving the growth rate differences between SGM and RGM, whose impact on growth rate warrants further confirmation with more studies.

## Data availability statement

The datasets presented in this study can be found in online repositories. The names of the repository/repositories and accession number(s) can be found in the article/Supplementary material.

## Author contributions

FC and CJ conceived the study. AL and OS provided valuable assistance in its adjustment. MZ, PW, and CL adjusted the bioinformatic workflow and conducting the analyses. MZ and PW created the figures. FC, MZ, PW, and CL wrote the manuscript. JW, XW, LY, XJ, and YS provided critical revisions and valuable insights during the writing process. All authors contributed to the article and approved the submitted version.

## Funding

This work was supported by the Funds for International Cooperation and Exchange of the National Natural Science Foundation of China (Grant no. 32061143024). AL is supported by the Israeli Science Foundation (Grants #1535/20, #3300/20, #3062/20).

## Conflict of interest

The authors declare that the research was conducted in the absence of any commercial or financial relationships that could be construed as a potential conflict of interest.

## Publisher’s note

All claims expressed in this article are solely those of the authors and do not necessarily represent those of their affiliated organizations, or those of the publisher, the editors and the reviewers. Any product that may be evaluated in this article, or claim that may be made by its manufacturer, is not guaranteed or endorsed by the publisher.

## Footnotes

^1^ https://www.ncbi.nlm.nih.gov/

## References

[ref1] Bachmann N. L. Salamzade R. Manson A. L. Whittington R. Sintchenko V. Earl A. M.. (2019). Key transitions in the evolution of rapid and slow growing mycobacteria identified by comparative genomics. Front. Microbiol. 10:3019. doi: 10.3389/fmicb.2019.03019 32038518 PMC6985099

[ref2] Bentley S. D. Parkhill J. (2004). Comparative genomic structure of prokaryotes. Annu. Rev. Genet. 38, 771–791. doi: 10.1146/annurev.genet.38.072902.094318 15568993

[ref3] Bieger B. Essen L. O. Oesterhelt D. (2003). Crystal structure of halophilic dodecin: a novel, dodecameric flavin binding protein from *Halobacterium salinarum*. Structure 11, 375–385. doi: 10.1016/S0969-2126(03)00048-0, PMID: 12679016

[ref4] Bouam A. Armstrong N. Levasseur A. Drancourt M. (2018). Mycobacterium terramassiliense, mycobacterium rhizamassiliense and mycobacterium numidiamassiliense sp. nov., three new *Mycobacterium simiae* complex species cultured from plant roots. Sci. Rep. 8:9309. doi: 10.1038/s41598-018-27629-1, PMID: 29915369 PMC6006331

[ref5] Brynildsrud O. Bohlin J. Scheffer L. Eldholm V. (2016). Rapid scoring of genes in microbial pan-genome-wide association studies with Scoary. Genome Biol. 17:238. doi: 10.1186/s13059-016-1108-8, PMID: 27887642 PMC5124306

[ref6] Devulder G. De Montclos M. P. Flandrois J. P. (2005). A multigene approach to phylogenetic analysis using the genus mycobacterium as a model. Int. J. Syst. Evol. Microbiol. 55, 293–302. doi: 10.1099/ijs.0.63222-0 15653890

[ref7] Edgar R. C. (2021). Muscle v5 enables improved estimates of phylogenetic tree confidence by ensemble bootstrapping. bioRxiv. 20.449169. doi: 10.1101/2021.06.20.449169

[ref8] Emms D. M. Kelly S. (2015). OrthoFinder: solving fundamental biases in whole genome comparisons dramatically improves orthogroup inference accuracy. Genome Biol. 16:157. doi: 10.1186/s13059-015-0721-2, PMID: 26243257 PMC4531804

[ref9] Eroshenko D. V. Polyudova T. V. Pyankova A. A. (2020). Vapbc and Mazef toxin/antitoxin systems in the regulation of biofilm formation and antibiotic tolerance in nontuberculous mycobacteria. Int J Mycobacteriol 9, 156–166. doi: 10.4103/ijmy.ijmy_61_20, PMID: 32474537

[ref10] Fedrizzi T. Meehan C. J. Grottola A. Giacobazzi E. Fregni Serpini G. Tagliazucchi S.. (2017). Genomic characterization of nontuberculous mycobacteria. Sci. Rep. 7:45258. doi: 10.1038/srep45258, PMID: 28345639 PMC5366915

[ref11] Gupta R. S. Lo B. Son J. (2018). Phylogenomics and comparative genomic studies robustly support division of the genus mycobacterium into an emended genus mycobacterium and four novel genera. Front. Microbiol. 9:67. doi: 10.3389/fmicb.2018.00067, PMID: 29497402 PMC5819568

[ref12] Hisanaga Y. Ago H. Nakagawa N. Hamada K. Ida K. Yamamoto M.. (2004). Structural basis of the substrate-specific two-step catalysis of long chain fatty acyl-CoA synthetase dimer. J. Biol. Chem. 279, 31717–31726. doi: 10.1074/jbc.M400100200, PMID: 15145952

[ref13] Hudson B. H. York J. D. (2012). Roles for nucleotide phosphatases in sulfate assimilation and skeletal disease. Adv Biol Regul 52, 229–238. doi: 10.1016/j.advenzreg.2011.11.002, PMID: 22100882 PMC3845023

[ref14] Hwang W. C. Bakolitsa C. Punta M. Coggill P. C. Bateman A. Axelrod H. L.. (2013). Lud, a new protein domain associated with lactate utilization. Bmc Bioinformatics 14:341. doi: 10.1186/1471-2105-14-341, PMID: 24274019 PMC3924224

[ref15] Ives A. R. Garland T. (2010). Phylogenetic logistic regression for binary dependent variables. Syst. Biol. 59, 9–26. doi: 10.1093/sysbio/syp074, PMID: 20525617

[ref16] Iyer L. M. Makarova K. S. Koonin E. V. Aravind L. (2004). Comparative genomics of the FtsK-HerA superfamily of pumping Atpases: implications for the origins of chromosome segregation, cell division and viral capsid packaging. Nucleic Acids Res. 32, 5260–5279. doi: 10.1093/nar/gkh828, PMID: 15466593 PMC521647

[ref17] Jia X. Yang L. Li C. Xu Y. Yang Q. Chen F. (2021). Combining comparative genomic analysis with machine learning reveals some promising diagnostic markers to identify five common pathogenic non-tuberculous mycobacteria. Microb. Biotechnol. 14, 1539–1549. doi: 10.1111/1751-7915.13815, PMID: 34019733 PMC8313281

[ref18] Johansen M. D. Herrmann J. L. Kremer L. (2020). Non-tuberculous mycobacteria and the rise of *Mycobacterium abscessus*. Nat. Rev. Microbiol. 18, 392–407. doi: 10.1038/s41579-020-0331-1 32086501

[ref19] Kashiwagi K. Miyamoto S. Nukui E. Kobayashi H. Igarashi K. (1993). Functions of potA and potD proteins in spermidine-preferential uptake system in *Escherichia coli*. J. Biol. Chem. 268, 19358–19363. doi: 10.1016/S0021-9258(19)36522-6, PMID: 8366082

[ref20] Kim C. J. Kim N. H. Song K. H. Choe P. G. Kim E. S. Park S. W.. (2013). Differentiating rapid- and slow-growing mycobacteria by difference in time to growth detection in liquid media. Diagn. Microbiol. Infect. Dis. 75, 73–76. doi: 10.1016/j.diagmicrobio.2012.09.019, PMID: 23114094

[ref21] Levy A. Salas Gonzalez I. Mittelviefhaus M. Clingenpeel S. (2018). Genomic features of bacterial adaptation to plants. Nat. Genet. 50, 138–150. doi: 10.1038/s41588-017-0012-9, PMID: 29255260 PMC5957079

[ref22] Lupoli T. J. Fay A. Adura C. Glickman M. S. (2016). Reconstitution of a *Mycobacterium tuberculosis* proteostasis network highlights essential cofactor interactions with chaperone DnaK. Proc. Natl. Acad. Sci. U. S. A. 113, E7947–e7956. doi: 10.1073/pnas.1617644113 PMID: 27872278 PMC5150378

[ref1001] Makarova K. S. Haft D. H. Barrangou R. Brouns S. J. Charpentier E. Horvath P.. (2011). Evolution and classification of the CRISPR-Cas systems. Nat. Rev. Microbiol. 9, 467–477. doi: 10.1038/nrmicro2577 21552286 PMC3380444

[ref23] Mann S. Chen Y. P. (2010). Bacterial genomic G+C composition-eliciting environmental adaptation. Genomics 95, 7–15. doi: 10.1016/j.ygeno.2009.09.002, PMID: 19747541

[ref24] Matsumoto Y. Kinjo T. Motooka D. Nabeya D. Jung N. Uechi K.. (2019). Comprehensive subspecies identification of 175 nontuberculous mycobacteria species based on 7547 genomic profiles. Emerg Microbes Infect 8, 1043–1053. doi: 10.1080/22221751.2019.1637702, PMID: 31287781 PMC6691804

[ref25] Mignard S. Flandrois J. P. (2008). A seven-gene, multilocus, genus-wide approach to the phylogeny of mycobacteria using supertrees. Int. J. Syst. Evol. Microbiol. 58, 1432–1441. doi: 10.1099/ijs.0.65658-0 18523191

[ref26] Peebo K. Valgepea K. Maser A. Nahku R. Adamberg K. Vilu R. (2015). Proteome reallocation in *Escherichia coli* with increasing specific growth rate. Mol. BioSyst. 11, 1184–1193. doi: 10.1039/C4MB00721B, PMID: 25712329

[ref27] Pereira A. C. Ramos B. (2020). Non-tuberculous mycobacteria: molecular and physiological bases of virulence and adaptation to ecological niches. Microorganisms 8:1380. doi: 10.3390/microorganisms8091380, PMID: 32916931 PMC7563442

[ref28] Price M. N. Dehal P. S. Arkin A. P. (2009). FastTree: computing large minimum evolution trees with profiles instead of a distance matrix. Mol. Biol. Evol. 26, 1641–1650. doi: 10.1093/molbev/msp077, PMID: 19377059 PMC2693737

[ref29] Prisic S. Husson R. N. (2014). *Mycobacterium tuberculosis* serine/threonine protein kinases. Microbiol Spectr 2:2013. doi: 10.1128/microbiolspec.mgm2-0006-2013, PMID: 25429354 PMC4242435

[ref30] Rathnapala J. Ragab W. Kawato S. Furukawa M. Nozaki R. Kondo H. (2023). Genomic characterization and identification of virulence-related genes in *Vibrio nigripulchritudo* isolated from white leg shrimp *Penaeus vannamei*. J. Fish Dis. 46, 779–790. doi: 10.1111/jfd.13786, PMID: 36989191

[ref32] Seemann T. (2014). Prokka: rapid prokaryotic genome annotation. Bioinformatics 30, 2068–2069. doi: 10.1093/bioinformatics/btu153, PMID: 24642063

[ref33] Shin M.-K. Park H.-T. Shin S. W. Jung M. Im Y. B. Park H.-E.. (2015). Whole-blood gene-expression profiles of cows infected with *Mycobacterium avium* subsp. paratuberculosis reveal changes in immune response and lipid metabolism. J. Microbiol. Biotechnol. 25, 255–267. doi: 10.4014/jmb.1408.08059, PMID: 25248984

[ref34] Šmarda P. Bureš P. Horov L. Leitch I. J. Mucina L. Pacini E.. (2014). Ecological and evolutionary significance of genomic Gc content diversity in monocots. Proc. Natl. Acad. Sci. U. S. A. 111, E4096–E4102. doi: 10.1073/pnas.1321152111, PMID: 25225383 PMC4191780

[ref35] Song L. Wu S. Tsang A. (2018). “Phylogenetic analysis of protein family” in Fungal genomics: Methods and protocols. eds. Vries R. P. Tsang A. Grigoriev I. V. (New York, Ny: Springer New York)

[ref36] Stinear T. P. Seemann T. Pidot S. Frigui W. Reysset G. Garnier T.. (2007). Reductive evolution and niche adaptation inferred from the genome of *Mycobacterium ulcerans*, the causative agent of Buruli ulcer. Genome Res. 17, 192–200. doi: 10.1101/gr.5942807, PMID: 17210928 PMC1781351

[ref37] Tan L. T.-H. Raghunath P. Ming L. C. Law J. W.-F. (2020). Mycobacterium ulcerans and *Mycobacterium marinum*: pathogenesis, diagnosis and treatment. Progress In Microbes & Molecular Biology 3:114. doi: 10.36877/pmmb.a0000114

[ref38] Thakur A. Mikkelsen H. Jungersen G. (2019). Intracellular pathogens: host immunity and microbial persistence strategies. J Immunol Res 2019:1356540. doi: 10.1155/2019/1356540 31111075 PMC6487120

[ref39] Tortoli E. (2012). Phylogeny of the genus mycobacterium: many doubts, few certainties. Infect. Genet. Evol. 12, 827–831. doi: 10.1016/j.meegid.2011.05.025, PMID: 21684354

[ref40] Tortoli E. Fedrizzi T. Meehan C. J. Trovato A. Grottola A. Giacobazzi E.. (2017). The new phylogeny of the genus mycobacterium: the old and the news. Infect. Genet. Evol. 56, 19–25. doi: 10.1016/j.meegid.2017.10.013 29030295

[ref41] Tran T. Bonham A. J. Chan E. D. Honda J. R. (2019). A paucity of knowledge regarding nontuberculous mycobacterial lipids compared to the tubercle bacillus. Tuberculosis (Edinb.) 115, 96–107. doi: 10.1016/j.tube.2019.02.008, PMID: 30948183

[ref42] Turenne C. Y. (2019). Nontuberculous mycobacteria: insights on taxonomy and evolution. Infect. Genet. Evol. 72, 159–168. doi: 10.1016/j.meegid.2019.01.017, PMID: 30654178

[ref43] Vitt S. Prinz S. Eisinger M. Ermler U. (2022). Purification and structural characterization of the Na(+)-translocating ferredoxin: Nad(+) reductase (Rnf) complex of *Clostridium tetanomorphum*. Nat. Commun. 13:6315. doi: 10.1038/s41467-022-34007-z, PMID: 36274063 PMC9588780

[ref9001] Wang X. Kim Y. Ma Q. Hong S. H. Pokusaeva K. Sturino J. M.. (2010). Cryptic prophages help bacteria cope with adverse environments. Nat. Commun. 1:147. doi: 10.1038/ncomms1146 21266997 PMC3105296

[ref44] Wee W. Y. Dutta A. Choo S. W. (2017). Comparative genome analyses of mycobacteria give better insights into their evolution. PLoS One 12:e0172831. doi: 10.1371/journal.pone.0172831, PMID: 28291784 PMC5349653

[ref45] Wright R. M. Yablonsky M. D. Shalita Z. P. Goyal A. K. Eveleigh D. E. (1992). Cloning, characterization, and nucleotide sequence of a gene encoding *Microbispora bispora* BglB, a thermostable beta-glucosidase expressed in *Escherichia coli*. Appl. Environ. Microbiol. 58, 3455–3465. doi: 10.1128/aem.58.11.3455-3465.1992, PMID: 1482172 PMC183129

[ref46] Wu H. Zhang Z. Hu S. Yu J. (2012). On the molecular mechanism of Gc content variation among eubacterial genomes. Biol. Direct 7:2. doi: 10.1186/1745-6150-7-2, PMID: 22230424 PMC3274465

