# Supplementary figures and images for: Comparative genomic analysis reveals differential genomic characteristics and featured genes between rapid- and slow-growing non-tuberculous mycobacteria
Source: Front Microbiol. 2023 Sep 21;14:1243371. doi: 10.3389/fmicb.2023.1243371 (PMC10551460; doi:10.3389/fmicb.2023.1243371)

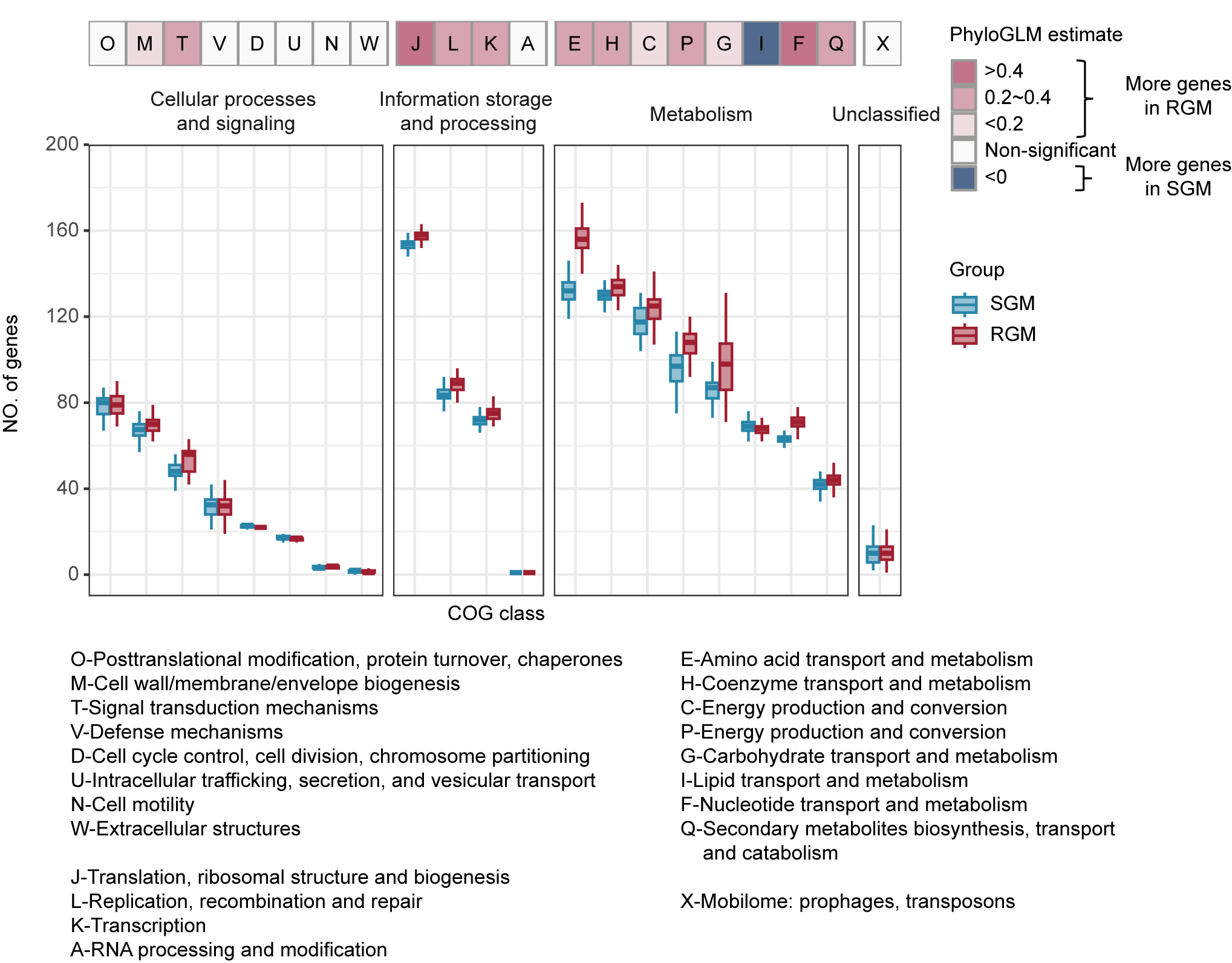

Supplement: Supplementary file 10 [file Image_1.TIF]

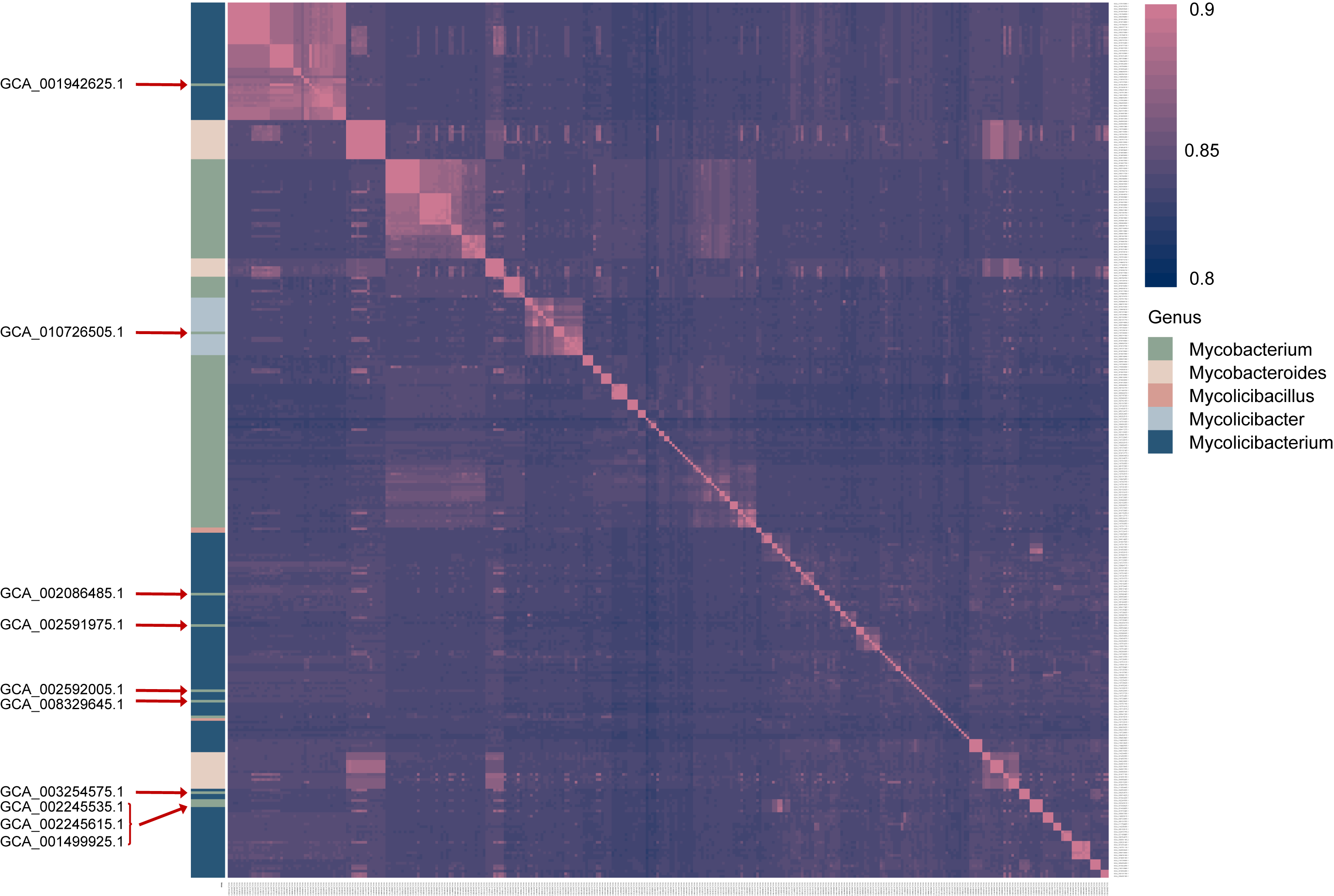

Supplement: Supplementary file 11 [file Image_2.TIF]

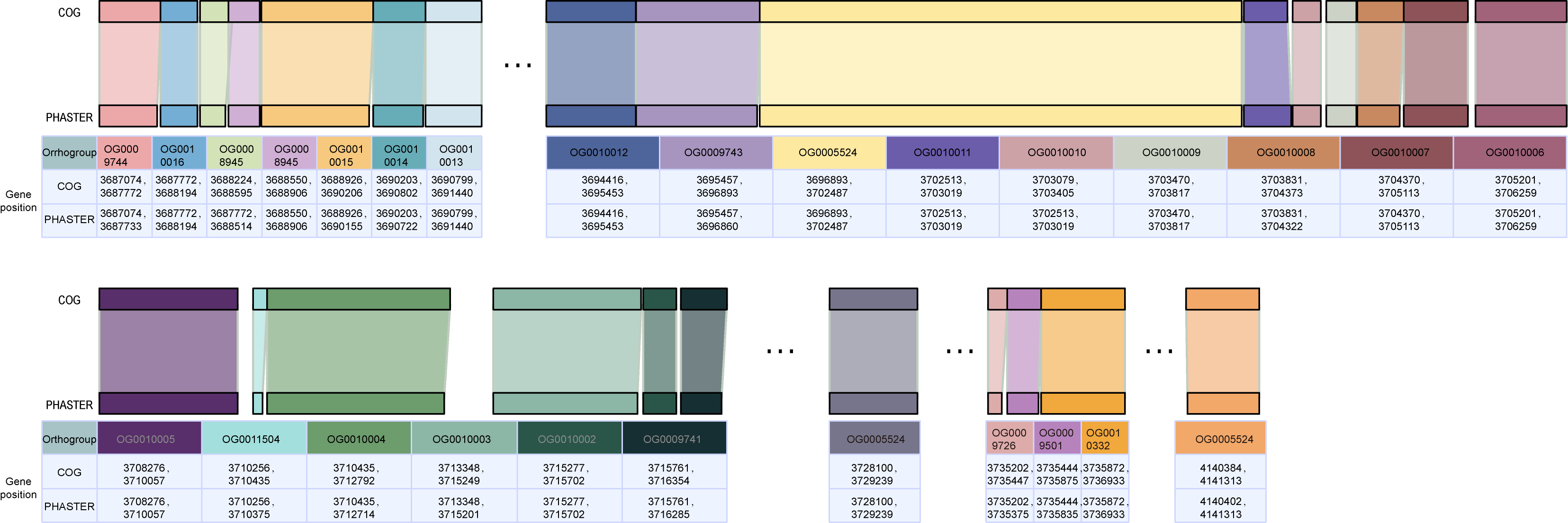

Supplement: Supplementary file 12 [file Image_3.TIF]
